# Closing the patient experience chasm: A two‐level validation of the Consumer Quality Index Inpatient Hospital Care

**DOI:** 10.1111/hex.12545

**Published:** 2017-02-20

**Authors:** Alina Smirnova, Kiki M. J. M. H. Lombarts, Onyebuchi A. Arah, Cees P. M. van der Vleuten

**Affiliations:** ^1^ Department of Educational Development and Research Maastricht University Maastricht The Netherlands; ^2^ Center for Evidence‐Based Education Academic Medical Center Amsterdam The Netherlands; ^3^ Department of Epidemiology UCLA Fielding School of Public Health Los Angeles USA; ^4^ Center for Health Policy Research UCLA Fielding School of Public Health Los Angeles USA

**Keywords:** Confirmatory factor analysis, generalizability theory, Consumer Quality Index (CQI), national surveys, patient‐centered care, quality assessment

## Abstract

**Background:**

Evaluation of patients’ health care experiences is central to measuring patient‐centred care. However, different instruments tend to be used at the hospital or departmental level but rarely both, leading to a lack of standardization of patient experience measures.

**Objective:**

To validate the Consumer Quality Index (CQI) Inpatient Hospital Care for use on both department and hospital levels.

**Design:**

Using cross‐sectional observational data, we investigated the internal validity of the questionnaire using confirmatory factor analyses (CFA), and the generalizability of the questionnaire for use at the department and hospital levels using generalizability theory.

**Setting and participants:**

22924 adults hospitalized for ≥24 hours between 1 January 2013 and 31 December 2014 in 23 Dutch hospitals (515 department evaluations).

**Main variable:**

CQI Inpatient Hospital Care questionnaire.

**Results:**

CFA results showed a good fit on individual level (CFI=0.96, TLI=0.95, RMSEA=0.04), which was comparable between specialties. When scores were aggregated to the department level, the fit was less desirable (CFI=0.83, TLI=0.81, RMSEA=0.06), and there was a significant overlap between *communication with doctors* and *explanation of treatment* subscales. Departments and hospitals explained ≤5% of total variance in subscale scores. In total, 4‐8 departments and 50 respondents per department are needed to reliably evaluate subscales rated on a 4‐point scale, and 10 departments with 100‐150 respondents per department for binary subscales.

**Discussion and conclusions:**

The CQI Inpatient Hospital Care is a valid and reliable questionnaire to evaluate inpatient experiences in Dutch hospitals provided sufficient sampling is done. Results can facilitate meaningful comparisons and guide quality improvement activities in individual departments and hospitals.

## 1 INTRODUCTION

Evaluation of patients’ health care experiences has become central to measuring quality in health care and, as a result, health care providers are more often held responsible for monitoring and improving patients’ care experiences.^1^ Patient care experiences reflect the degree to which care is patient‐centred (ie care that is respectful and responsive to patients’ preferences, needs and values).^2^ In addition to its intrinsic value as an indicator of quality, a growing body of evidence points to the positive associations between positive patient experiences and clinical processes of care^3^, ^4^ as well as better patient adherence to treatment, improved clinical outcomes and decreased utilization of health care services.^5^

Even though improving patient care experiences is increasingly being incorporated in both local and global health agendas,^6^ patient feedback remains largely underutilized in local hospital improvement plans.^7^ One of the main reasons for this is lack of specific and timely feedback that is easily translatable to improvements on the frontline.^8^, ^9^ Current instruments used to collect patient experience data mostly collect data on hospital‐wide level for identification of larger national trends and contracting of hospital services. In order to bridge the gap between external reporting and internal quality assurance, some have recommended to use different instruments for different purposes.^9^, ^10^ This is, however, not desirable due to lack of standardization of measures, a lack of common language and possible disconnect between local improvement efforts and hospital‐wide measurements. Implementation of instruments is also costly and can potentially lead to duplication of work and unnecessary use of valuable resources.

An alternative approach is to adapt existing instruments to reflect their multiple purposes. In this study, we attempt to address these problems using the Dutch version of the American Hospital Consumer Assessment of Healthcare Providers and Systems (HCAHPS) survey, which was imported into the Netherlands in 2006 by Arah et al. for use within the Dutch health care system.^11^ This has led to the development of nationally used standardized questionnaires and protocols called the Consumer Quality Index (CQI), wherein the Dutch HCAHPS is known as the CQI Inpatient Hospital Care.^12^ Efforts to adapt this questionnaire for multiple purposes, including external accountability and internal quality assurance, have resulted in different versions of the questionnaire to be produced.^13^, ^14^ However, no extensive validation of the CQI Inpatient Hospital Care has occurred since the original validation study by Arah et al. (2006). As the results are consequentially used by patients, hospital staff, health insurers, the inspectorate and researchers for different purposes, it is imperative that the questionnaire can evaluate and differentiate patient care experiences across hospitals, specialties as well as departments reliably and validly. We aimed to assess the internal validity and reliability of the CQI Inpatient Hospital Care on both hospital and department levels. Additionally, we investigated whether the questionnaire measured similar domains of patient experiences across four specialties, namely surgery, obstetrics and gynaecology, internal medicine and cardiology.

## 2 METHODS

### 2.1 Setting and study population

We analysed CQI Inpatient Hospital Care questionnaire data from 23 Dutch hospitals including four academic centres, 515 department evaluations in 17 specialties (nine surgical and eight medical) collected between 1 January 2013 and 31 December 2014. Eligible patients 16 years or older who were hospitalized for at least 24 hours with a discharge within the previous 12 months were identified using hospital admission lists. Eligible participants were invited to evaluate their experiences of hospitalization using either online or paper‐based CQI Inpatient Hospital Care (Appendix S1). Evaluations collected in 2013 were used for national benchmarking among 43 hospitals in four specialties, namely surgery, internal medicine, cardiology, obstetrics and gynaecology. Therefore, we focused on these specialties in this study. The hospitals and clinical departments that re‐evaluated their inpatient hospital care using the same questionnaire in 2014 for own internal quality assurance purposes were considered to be independent evaluations and were, therefore, also included in the analysis. We analysed the results both for 2013 and 2014 together and separately, and if there was no change, reported the combined results only.

As retrospective research does not fall under the Dutch Medical Research Involving Human Subjects Act (WMO), an official ethical review was not required for this study. Nonetheless, we obtained permissions from individual hospitals to use anonymized questionnaire data for research purposes. Furthermore, we consulted a privacy officer at our institution to ensure that the data provided for this research complied with Dutch Personal Data Protection Act. Participating hospitals were recruited through the Miletus Foundation (www.stichtingmiletus.nl), a coordinating body of all CQI evaluations within the Netherlands. A detailed research proposal was sent to all hospitals and subsequently discussed at the general meeting. Hospitals interested in participating in the study gave informed consent either via the Miletus Foundation or by directly contacting the primary researcher (AS). MediQuest (home.mediquest.nl), a company that processes patient evaluation data from these evaluations, provided the final data set for the study.

### 2.2 CQI Inpatient Hospital Care questionnaire

The CQI Inpatient Hospital Care questionnaire has been developed in co‐operation with patient and consumer organizations based on three existing instruments used to measure patient care experiences: the CAHPS Hospital Care questionnaire, the Dutch Hospital Association inpatient satisfaction questionnaire and the Hospital Comparison questionnaire from the Netherlands Institute for Health Services Research and the Consumers’ Association.^13^, ^15^ The CQI Inpatient Hospital Care consists of a total of 50 items: 38 items about patient experiences and 12 items asking background information. An earlier exploratory factor analysis^14^ identified nine domains of patient experience, namely *admission* (Q4a‐j), *communication with nurses* (Q6‐8), *communication with doctors* (Q9‐10), *own contribution* (Q13‐15, 17, 25), *explanation of treatment* (Q18‐20), *pain management* (Q21‐22), *communication about medication* (Q23‐24), *feeling of safety* (Q27‐29) and *discharge information* (Q31‐34). *Admission* and *information at discharge* were assessed on a 2‐point scale (yes=1, no=0). Other scales were assessed on a 4‐point Likert scale ranging from 1 (Never) to 4 (Always). Building on this previously identified work, we used this structure to test the internal validity, reliability and generalizability of the CQI Inpatient Hospital Care questionnaire.

### 2.3 Statistical analysis

First, respondents and non‐respondents were described using descriptive statistics. Questionnaires were excluded if they had a negative or no response to the question whether or not the patient had a hospital admission within the last 12 months or if less than half of core items were completed. Evaluations with missing data were imputed using multiple imputation technique to create 10 complete data sets.^16^ Multiple imputation was preferable to single‐imputation methods such as maximum‐likelihood approaches because it better reflected the inherent uncertainty due to missing data in the sample.^17^ Convergence of the imputations was assessed by examining trace plots and calculating the *Rhat* statistic.^18^ In order to maximize convergence, we increased the number of maximum iterations to 200. We then calculated the subscale scores for each imputed data set by averaging the scores for the items within each subscale.

The internal validity of the questionnaire was evaluated by assessing the fit of the pre‐identified 9‐factor structure of the questionnaire. In order to assess the overall fit of the model, we performed a confirmatory factor analysis (CFA) on all imputed data sets and combined the final results using Rubin's rules. For categorical variables, weighted least squares with mean and variance adjusted (WLSMV) estimator was preferred to account for the categorical nature of the answers. The WLSMV estimator is a robust estimator that does not assume normally distributed variables and is preferred for modelling categorical or ordered data.^19^ We assessed the global model fit using the comparative fit index (CFI), Tucker‐Lewis index (TLI) and root mean square error of approximation (RMSEA).^20^ The following cut‐off values indicated a good fit: CFI≥0.95, TLI≥0.95 and RMSEA≤0.06.^19^ The overall fit was deemed acceptable if at least two of the three criteria of fit indices were met.^21^ In order to establish whether the questionnaire measured similar patient experiences across various medical specialties, CFA was then repeated in four subgroups: surgery, obstetrics and gynaecology, internal medicine and cardiology. These specialties were chosen because these specialties were included in the national benchmark. Same cut‐off points were used to evaluate the fit of the factor structure as for the overall sample. Finally, we repeated the CFA on the department level by aggregating the scores of each variable to the department level.

Internal consistency of the subscales was evaluated by calculating Cronbach's α statistic for individual questionnaires and the department in each imputed data set, and averaging it across imputed data sets. Overall Cronbach's α≥0.70 was deemed acceptable. The degree to which the subscales measured distinct concepts was assessed by calculating inter‐scale correlations, which were also calculated for individual scores and scores aggregated to the department. A correlation of <0.70 indicated that there was no significant overlap between the subscales. Construct validity was assessed by examining the relative importance of the subscales with two global ratings, namely overall evaluation of the department (Q36, scale 0‐10) and hospital (Q35, scale 0‐10) using multiple linear regression and accounting for respondents’ age, sex, education, self‐rated physical health, self‐rated psychological health, country of origin and the number of admissions in the previous 12 months.

Generalizability analysis was conducted to estimate the minimum number of respondents needed to reliably evaluate each subscale on both department and hospital levels. For department‐level evaluations, we estimated a model where the number of items was considered as fixed, with department (*d*) as the unit of analysis, where respondents (*p*) were nested within departments (*p:d*). The resulting design was unbalanced single‐facet nested design.^22^ For hospital‐level analyses, we similarly regarded the number of items as fixed; however, this time we regarded hospital as the unit of analysis and respondents (*p*) to be nested within departments (*d*), which were, in turn, nested within hospitals (h), resulting in an multifacet unbalanced nested design (*p:d:h*). We averaged variance components, including variance across the departments (S_d_) and respondents nested within departments (S_p:d_) and respondents nested within departments and hospitals (S_p:d:h_), across imputed data sets. Then, we estimated the proportion of the total variance in scores that are due to differences between departments or hospitals. In a D‐study, we estimated the G coefficient and the standard error of measurement (SEM) associated with varying number of respondents within departments and departments within hospitals for mean subscale scores. For seven scales evaluated on a 4‐point scale, we used 0.4 units as an admissible level of “noise,” representing SEM<0.10 (1.96×0.10×2≈0.4) as the maximum value for 95% confidence interval interpretation. For dichotomous scales, we used 0.1 on a scale of 0‐1 as an admissible level of noise, representing SEM<0.025 (1.96×0.025×2≈0.1).

Missing data were imputed using the *mice* package (version 2.25) in *R* statistical software version 3.2.3.^23^, ^24^ The confirmatory factor analyses on imputed data sets were performed using the *semTools* package (version 0.4‐11) and on aggregated data sets using the *lavaan* package (version 0.5‐20) in *R* version 3.2.3.^25^ Inter‐scale correlations, Cronbach's α, variance components calculations and multiple linear regression analyses were performed using SPSS version 23.0.0.2 (IBM SPSS Inc., Chicago, IL, USA).

## 3 RESULTS

Of the distributed 74090 questionnaires, 23476 were returned (gross response rate 31.7%). Table 1 reports characteristics of respondents and non‐respondents. In total, 552 questionnaires were excluded due to negative or no response to the question whether or not they had a hospital admission within the last 12 months or less than half of core items completed. The resulting sample size was 22924 (net response rate 30.9%), including 23 hospitals, 17 different specialties and 515 department evaluations. Table 2 further describes the demographic characteristics of the included respondents. As the results did not differ between 2013 and 2014, we report only combined results below.

**Table 1:** Characteristics of respondents and non‐respondents of the CQI Inpatient Hospital Care questionnaire

Characteristic	Respondents N (%) (n=23 476)	Non‐respondents N (%) (n=50 614)	Total N (%) (n=74 090)
Gender			
Male	11 255 (47.9)	21 802 (43.1)	33 057 (44.6)
Female	12 221 (52.1)	28 812 (56.9)	41 033 (55.4)
Age (years)			
16‐24	486 (2.1)	3623 (7.2)	4109 (5.5)
25‐34	1580 (6.7)	6999 (13.8)	8579 (11.6)
35‐44	1833 (7.8)	6356 (12.6)	8189 (11.1)
45‐54	3062 (13.0)	7246 (14.3)	10 308 (13.9)
55‐64	5195 (22.1)	8224 (16.2)	13 419 (18.1)
65‐74	5492 (23.4)	9720 (19.2)	15 212 (20.5)
75‐79	2737 (11.7)	3088 (6.1)	5825 (7.9)
80+	3091 (13.2)	5358 (10.6)	8449 (11.4)
Type of questionnaire			
Online	17 922 (76.3)	‐	‐
Mail	5554 (23.7)	‐	‐

**Table 2:** Characteristics of the respondents included in validation of the CQI Inpatient Hospital Care questionnaire

Characteristic	N (Total=22 924)	%
Gender		
Male	10 992	47.9
Female	11 932	52.1
Age (years)		
16‐24	486	2.1
25‐34	1572	6.9
35‐44	1828	8.0
45‐54	3053	13.3
55‐64	5170	22.6
65‐74	5462	23.8
75‐79	2535	11.1
80+	2818	12.3
Level of education		
Lower secondary or less	6561	28.6
Upper secondary	10 511	45.9
Tertiary	5852	25.5
Self‐reported health		
Excellent	1389	6.1
Very good	2962	21.9
Good	10 673	46.6
Average	6694	29.2
Bad	1206	5.3
Self‐reported psychological health		
Excellent	4149	18.1
Very good	5460	23.8
Good	10 968	47.8
Average	2130	9.3
Bad	217	0.9
Country of origin		
The Netherlands	21 152	92.3
Germany	156	0.7
(Former) Netherlands Antilles/Aruba/Suriname	293	1.3
Indonesia/Netherlands Indies	281	1.2
Morocco/Turkey	194	0.8
Other	738	3.2
Missing	110	0.5
Number of admissions in the previous 12 months including current one		
1	13 283	57.9
2	5947	25.9
3	2119	9.2
4+	1464	6.4
Missing	111	0.5
Specialty		
Surgical	11 344	49.5
General surgery	3225	14.1
Orthopaedic surgery	2502	10.9
Urology	1773	7.7
Cardiothoracic surgery	895	3.9
Neurosurgery	822	3.6
Otolaryngology	743	3.2
Obstetrics and gynaecology	643	2.8
Plastic surgery	607	2.6
Ophthalmology	134	0.6
Medical	8000	34.9
Cardiology	2697	11.8
Internal medicine	1984	8.7
Pulmonology	1877	8.2
Neurology	1262	5.5
Rheumatology	67	0.3
Geriatrics	54	0.2
Dermatology	38	0.2
Anaesthesiology	21	0.1
Missing	3580	15.6

### 3.1 Psychometric properties

CFA showed a good fit for surgical, obstetrics and gynaecology, internal medicine, cardiology specialties and all specialties combined (Table 3). When the scores were aggregated to the department level, the incremental fit indices decreased to CFI=0.83 and TLI=0.81. Internal consistency of the scales was acceptable, except for subscales *own contribution* (0.69), *communication about medication* (0.68) and *feeling of safety* (0.64). On the department level, all subscales demonstrated acceptable Cronbach's α, except for *feeling of safety* (0.64) (Table 4). Inter‐scale correlations showed that on the department level, the subscales *communication with doctors* with *explanation of treatment* overlapped substantially (Pearson's *r*=0.72) (Table 4). *Communication of treatment* did not predict global ratings of either the hospital or the department, while *explanation of treatment* was a significant predictor of the rating of the hospital but not the global rating of the department (Table 4).

**Table 3:** Fit indices for surgery, cardiology, internal medicine, and obstetrics and gynaecology, and all specialties on individual (patient) level and department level. Department‐level scores were obtained by calculating the means for every item per department across all imputed data sets

	Surgery (n=3225) Individual level	Cardiology (n=2697) Individual level	Internal medicine (n=1984) Individual level	Obstetrics and gynaecology (n=643) Individual level	All specialties (n=22 924) Individual evel	All specialties (n=515) Department level
CFI (≥0.95)	0.98	0.97	0.98	0.98	0.96	0.83
TLI (≥0.95)	0.98	0.96	0.98	0.97	0.95	0.81
RMSEA (≤0.06)	0.03	0.03	0.03	0.02	0.04	0.06

**Table 4:** Scale means with standard deviations (SD), reliability coefficients (Cronbach's α) and inter‐scale correlations, for individual (above the diagonal) and aggregated department (below the diagonal) evaluations, and estimates of multiple linear regression analyses with 95% confidence intervals examining associations with global department and hospital ratings corrected for respondents’ age, sex, level of education, self‐rated physical and psychological health, number of admissions in the previous 12 mo, and place of birth (*P≤.05, **P≤.001)

Subscale (scoring range)	Mean (SD)	Cronbach's α individual (department)	Inter‐scale correlations									Global rating department	Global rating hospital
			1	2	3	4	5	6	7	8	9		
*1. Admission (0‐1)*	0.6 (0.25)	0.77 (0.81)	1	0.30	0.29	0.31	0.39	0.25	0.38	0.35	0.42	0.14 (0.05‐0.23)*	0.20 (0.12‐0.27)**
*2. Communication with nurses (1‐4)*	3.4 (0.61)	0.83 (0.87)	0.36	1	0.56	0.49	0.51	0.55	0.47	0.46	0.35	1.00 (0.97‐1.04)**	0.59 (0.56‐0.62)**
*3. Communication with doctors (1‐4)*	3.4 (0.71)	0.81 (0.84)	0.41	0.56	1	0.42	0.56	0.41	0.43	0.37	0.33	0.08 (0.05‐0.11)**	0.27 (0.25‐0.30)**
*4. Own contribution (1‐4)*	3.0 (0.66)	0.69 (0.80)	0.31	0.50	0.47	1	0.44	0.38	0.46	0.39	0.31	0.31 (0.28‐0.34)**	0.27 (0.25‐0.30)**
*5. Explanation of treatment (1‐4)*	3.5 (0.67)	0.81 (0.89)	0.50	0.59	0.72	0.52	1	0.45	0.57	0.43	0.43	−0.03 (‐0.06‐0.00)	0.08 (0.05‐0.11)**
*6. Pain management (1‐4)*	3.5 (0.62)	0.79 (0.86)	0.48	0.68	0.52	0.42	0.61	1	0.42	0.41	0.32	0.34 (0.31‐0.38)**	0.26 (0.22‐0.29)**
*7. Communication about medication (1‐4)*	3.0 (0.91)	0.68 (0.85)	0.41	0.60	0.60	0.55	0.67	0.52	1	0.47	0.45	−0.01 (−0.03‐0.02)	0.004 (‐0.02‐0.03)
*8. Feeling of safety (1‐4)*	3.4 (0.68)	0.64 (0.64)	0.47	0.47	0.48	0.30	0.50	0.61	0.52	1	0.38	0.21 (0.18‐0.24)**	0.18 (0.15‐0.21)**
*9. Information at discharge (0‐1)*	0.7 (0.31)	0.76 (0.82)	0.60	0.49	0.50	0.43	0.60	0.50	0.53	0.42	1	0.54 (0.48‐0.60)**	0.52 (0.47‐0.57)**

5% or less of total variance in scores was attributable to the department or the hospital (Table 5). Results of the generalizability analysis showed that a minimum of 50 respondents is needed to reliably evaluate subscales of patient experience scored 1‐4 in a department (Appendix S2). For subscales evaluated on Yes/No (0‐1) scale (*admission* and *discharge information*), 100 and 150 patient evaluations were needed, respectively, for department‐level evaluations. For hospital‐level evaluations, subscales rated 1‐4 can reliably be evaluated with 4‐8 departments with at least 50 patient evaluations each. For *admission* and *discharge information*, at least 10 departments with 100 patient evaluations are needed.

**Table 5:** Variance components for departments, hospitals and residual variance

	Residual variance	Between‐department variance (% total variance)	Between‐hospital variance (% total variance)	Hospital variance vs hospital and department variance
*1. Admission*	0.059	0.003 (5%)	0.000 (0%)	0.0
*2. Communication with nurses*	0.360	0.005 (1%)	0.004 (1%)	0.44
*3. Communication with doctors*	0.490	0.006 (1%)	0.004 (1%)	0.40
*4. Own contribution*	0.404	0.014 (3%)	0.020 (5%)	0.59
*5. Explanation of treatment*	0.435	0.012 (3%)	0.003 (1%)	0.20
*6. Pain management*	0.376	0.008 (2%)	0.002 (1%)	0.20
*7. Communication about medication*	0.805	0.012 (1%)	0.008 (1%)	0.40
*8. Feeling of safety*	0.446	0.010 (2%)	0.002 (0%)	0.17
*9. Information at discharge*	0.089	0.005 (5%)	0.000 (0%)	0.0

## 4 DISCUSSION

To our knowledge, this is the first study to validate an inpatient experience questionnaire for multiple purposes, namely on the level of the hospital and the department. The CFA results showed a good overall fit, which was comparable between specialties. On the department level, however, the CFA showed a less desirable fit with a significant overlap on the department level between the subscales *communication with doctors* and *explanation of treatment*. Differences between departments and hospitals explained only a small proportion of total variance in patient experience scores, with the hospital and the department varying in importance depending on the subscales. A total of 4‐8 departments and 50 respondents per department are needed to reliably evaluate most subscales on both department and hospital levels. For binary subscales, such as *admission* and *discharge information*, a minimum of 100‐150 patients per department and 10 departments are needed.

The overall good fit provides evidence of validity for the internal structure of the CQI Inpatient Hospital Care questionnaire on the level that it was first designed for, that is the patient. The goodness‐of‐fit indices for surgery, obstetrics and gynaecology, cardiology and internal medicine specialties were similarly good, suggesting that patients experience similar aspects of care in different specialties, allowing for comparisons of patient experiences between specialties to be made. Previous research has demonstrated that, even though aspects of patient experience may be comparable across specialties, their importance can differ substantially by type of hospitalization.^26^ Although we did not research the relative importance of these aspects for different specialties, departments or hospitals will need to take this into account when choosing priorities for areas of quality improvement.

The internal consistency of the scales was acceptable except for three subscales: *own contribution*,* communication about medication* and *feeling of safety*. The same subscales also demonstrated a lower internal consistency in a previous pilot validation study.^14^ Furthermore, our study found that the subscale *communication about medication* did not significantly contribute to the global ratings of the department or the hospital, which may indicate a need for improvement in external validity of this scale. Alternatively, global ratings may not be a good indicator of overall health care quality and should, therefore, not be used in external validation, as research by Krol et al. has shown it may be measuring a different concept.^27^ Similar to other studies^11^, ^26^, ^28^, we found that *communication with nurses* was the strongest predictor of overall ratings of the department as well as the hospital. This is not surprising as nurses are the primary providers of care in the hospital environment. Furthermore, research has shown that factors related to nursing work such as nursing work environment, nurse‐to‐patient ratios^28^ and missed nursing care^29^ and nurse‐patient interaction^30^ can influence patient satisfaction ratings. A new finding, however, is that higher scores on the subscale *discharge information* significantly contributed to patients’ global ratings of both the hospital and the department. This is different from the findings by Elliott et al.^26^, in which discharge information was one of the least valued aspects of inpatient care and was important for only half of hospitalization types. This is not surprising as there appears to be a gap in communication between patients and providers at discharge. A survey of hospitalized patients showed that more than half of patients 70 years or older did not receive instructions about how to care for themselves after hospitalization.^31^ Our findings suggest that discharge information may be more important than previously thought and that hospitals and departments may improve the overall patient experience by improving how they handle discharges. Yet, as De Boer et al. demonstrated, although global ratings represent experiences regarding priorities, experiences with the important elements of care may still have inconsistent relationships with global ratings.^32^

On the department level, fit indices did not demonstrate an acceptable fit based on the incremental fit indices (CFI=0.83 and TLI=0.81), while RMSEA was within acceptable bounds at 0.06 (≤0.06 acceptable). As two of the three criteria do not meet the cut‐off criteria, we conclude that the current model is not a good representation of the latent constructs on the department level. Combined with the significant overlap between subscales *explanation of treatment* and *communication with doctors*, these results point that on the department level a different structure would provide a better fit of the data. Another reason for a poor fit of the structure on the department level could be the use of aggregated scores, which does not consider the variability of the scores within each department. This may have unnecessarily distorted the data. As the patients are naturally nested within departments and hospitals, confirmation of the fit using multilevel CFA is desirable.

The results of the variance component analysis showed that the department and the hospital each account for 5% or less in total variability of the subscale scores. This corresponds with previous research that has found limited influence of the department and the hospital on variability of patient experience scores.^15^, ^33^ Generalizability analysis found that it is possible to reliably evaluate patients’ experience using subscales with the scoring scale 1‐4 with 50 respondents (in 4‐8 departments for hospital‐level evaluations), and with 100‐150 respondents (in 10 departments) for the two subscales with the Yes/No (0‐1) scoring scale. More respondents are needed for binary subscales because of the small range of possible scores, leading to higher precision and reliability needed to detect small changes. Compared with other instruments,^5^, ^10^ this study shows an improvement in the number of respondents that are needed for reliable evaluation of patient experiences of a single department. Similar size samples are required to reliably evaluate all subscales on the hospital level using our criteria. However, different cut‐off criteria may be chosen depending on whether the results of the CQI Inpatient Hospital Care are to be used by departments for their own quality improvement purposes, or by health insurance companies and health‐care authorities to make summative judgements about the quality of care.^34^ We, therefore, recommend using the generalizability results of this study (shown in Appendix S2) to adjust the cut‐off criteria based on the proposed use of the questionnaire.

In interpreting the results, several limitations should be mentioned. Patient surveys suffer from low response rates. Our response rate of 31% was similar to those previously seen in this setting.^14^ Reasons for non‐response were not collected during the original data collection process, which made a non‐responder analysis impossible. Although we tried to account for non‐respondents by including sex and age as covariate in regression analyses, this may not have been sufficient because respondents and non‐respondents may also vary based on other characteristics that we have not been able to account for, such as country of origin, language spoken at home or level of education. For example, we did not have any data on how many patients were invited to fill out online or paper‐based questionnaire. Furthermore, in this study we aggregated the individual scores to the level of the department, because this is how typically the scores may be used. Other methods can be tried, such as using median or factor scores, but these may be difficult to interpret. Also, we did not test alternative models on the department level or factor equivalency between different specialties or respondents groups. Finally, we did not investigate the external validity of this questionnaire by studying the relationship between aspects of inpatient hospital care and other important process or outcome measures. Nonetheless, this study also has several strengths. One strength of this study is its use of more than 22000 patient evaluations and over 500 department evaluations from multiple specialties in multiple hospitals including academic and non‐academic centres, which supports the generalizability of our results. Another strength in this study is the use of multiple imputation for handing missing data, which accounts for the uncertainty associated with imputation of missing data.^17^

With this study, we contribute evidence for validity of the CQI Inpatient Hospital Care questionnaire and its utility for use in different settings and for both quality assurance and summative purposes. We recommend that stakeholders including hospitals, clinical departments and health insurers using this questionnaire use appropriate sample sizes based on its purpose and level of use. Considering the response rate is 31%, much larger samples may be required to arrive at recommended numbers of evaluations. Low response rates have become worrisomely common in survey research,^35^ with many studies now reporting rates as low as or lower than ours.^36^ Low response rates may indicate low levels of receptivity of the instrument by patients. Improvements in response rates, for example by identifying and addressing reasons for non‐response, are needed to ensure optimal use of resources as well as appropriate sample sizes. Although this questionnaire has originally been imported to facilitate standardization of the instrument for international comparisons,^11^ at this point, both the CQI Inpatient Hospital Care and the American HCAHPS, on which the CQI Inpatient Hospital Care is based, have changed substantially such that any international comparisons can only be made based on a collection of limited number of questions that are present in both questionnaires. Future research can investigate whether patient experiences of hospital care improve over time with continuous measurement. Like Zuidgeest et al.^37^ and Damman et al.*,* 38 we recommend using multilevel models for longitudinal and hierarchical data analyses, rather than using average department or hospital scores.

In conclusion, the CQI Inpatient Hospital Care questionnaire can provide valid and reliable data on patient experiences of inpatient hospital care on both department and hospital. The resulting data can be used to facilitate meaningful comparisons and guide quality improvement activities. Future research can focus on improving reliability of the scales, wording of the individual items to reflect specific provider or clinical settings better, and validating the structure on the department level and for different specialties.

## CONFLICTS OF INTEREST

All authors express no potential conflicts of interest.
