# Supplementary material for: Closing the patient experience chasm: A two‐level validation of the Consumer Quality Index Inpatient Hospital Care
Source: Health Expect. 2017 Feb 20;20(5):1041–8. doi: 10.1111/hex.12545 (PMC5600232; doi:10.1111/hex.12545)
Supplement: Supplementary file 1 [file HEX-20-1041-s001.doc]

Appendix 1. Original domains and associated items in the CQI Inpatient Hospital Care questionnaire (version 1.3).

| *Domain* | *Item No.* | *Description* | *Response categories* |
| --- | --- | --- | --- |
| *Hospital accessibility* | 2 | Has the accessibility of the hospital using your own means of transport been a problem? | Major problem;  Minor problem;  No problem;  Not applicable |
|  | 3 | Have the number of parking spaces at the hospital been a problem? | Ditto |
| *Admission* |  | Were the following items discussed with you on your admission to the hospital? |  |
|  | 4a | Your rights as patient (complaint procedure, etc.) | No;  Yes;  Do not remember |
|  | 4b | What will happen during this hospitalization | Ditto |
|  | 4c | A card or film on patient safety | Ditto |
|  | 4d | The person in the hospital whom you can contact if you have questions | Ditto |
|  | 4e | What medications you are taking | Ditto |
|  | 4f | If you would like to be resuscitated | Ditto |
|  | 4g | Any dietary and nutritional requirements | Ditto |
|  | 4h | Any hypersensitivity to substances/medication | Ditto |
|  | 4i | Your provisional discharge date | Ditto |
|  | 4j | Your personal needs during the hospitalization | Ditto |
|  | 5 | Did the healthcare providers/ staff, upon arrival to the ward, have enough time for you? | No;  Yes |
| *Nurses’ care during your hospitalization* | 6 | Did the nurses listen carefully to you? | Never;  Sometimes;  Mostly;  Always |
|  | 7 | Did the nurses have enough time for you? | Ditto |
|  | 8 | Did the nurses explain things to you in an understandable way? | Ditto |
| *Doctors’ care* | 9 | Did the doctors have enough time for you? | Ditto |
|  | 10 | Did the doctors explain things to you in an understandable way? | Ditto |
|  | 11 | Did the doctors give you conflicting information? | Ditto |
| *Your stay at the hospital* | 12 | Were the toilet, the shower and the bathroom in or near your room clean? | Ditto |
|  | 13 | Did you have enough privacy during your personal care? | Ditto |
|  | 14 | Could receive visitors at times that you wanted? | Ditto |
|  | 15 | Could you retire to a quiet place if you wanted to? | Ditto |
|  | 16 | Was the food tasty? | Ditto |
|  | 17 | Could you eat at the times that you wanted to? | Ditto |
| *Communication around treatment* | 18 | Did the doctors or nurses tell you beforehand why the treatment, examination or intervention was needed? | Ditto |
|  | 19 | Did the doctors or nurses tell you beforehand what the treatment, examination or intervention was? | Ditto |
|  | 20 | Did the doctors or nurses explain to you in an understandable way about any possible side effects or consequences of a treatment, examination or intervention? | Ditto |
|  | 21 | Did the staff at the hospital react quickly when you indicated you had pain? | Never;  Sometimes;  Mostly;  Always;  Not applicable, I never indicated to have had pain 🡪 go to question 23 |
|  | 22 | Was your pain well controlled? | Never;  Sometimes;  Mostly;  Always |
|  | 23 | Before you received a new medication or before your medication was changed, did you get an explanation of what was the purpose of the new medication? | Never;  Sometimes;  Mostly;  Always;  Not applicable, I do not use any medications 🡪 go to question 25 |
|  | 24 | Before you received a new medication or before your medication was changed, did you get an explanation of the possible complications in an understandable way? | Never;  Sometimes;  Mostly;  Always |
|  | 25 | During admission to the hospital how often did you have a say in matters that were important to you? | Ditto |
|  | 26 | Was the coordination of work between staff a problem? | A big problem;  A small problem;  No problem |
| *Safety in this hospital* | 27 | When medication was provided, did staff check whether the medication was intended for you, for example by asking your name or checking your wristband? | Never;  Sometimes;  Mostly;  Always;  I do not know (any more);  Not applicable, I did not use any medications |
|  | 28 | Before a treatment, examination, or intervention began, did staff check that you were the right person, for example by asking your date of birth? | Never;  Sometimes;  Mostly;  Always;  I do not know (any more) |
|  | 29 | Did the staff of the hospital pay enough attention to unsafe situations? | Never;  Sometimes;  Mostly;  Always; |
| *Discharge from the hospital* | 30 | Upon discharge from the hospital, did you receive written and verbal information about the use of new medications in combination with medication that you were already using? | No;  Yes;  I do not know (any more);  Not applicable, I did not use any medications before my hospitalization |
|  | 31 | Upon discharge from the hospital, did you receive information about submitting any complaints or health problems that you were supposed to be on the lookout for? | No;  Yes;  I do not know (any more) |
|  | 32 | Upon discharge from the hospital, did you receive information about which activities you could or could not do? | No;  Yes;  I do not know (any more) |
|  | 33 | Before you were discharged from the hospital, did you speak to the hospital staff about the help you might need after your discharge? | No;  Yes;  I do not know (any more) |
|  | 34 | Upon discharge from the hospital, did you get information about what to do if problems occurred after your discharge? | No;  Yes;  I do not know (any more) |

Appendix 2. G coefficients and standard error of measurement (SEM) for varying numbers of respondents for department-level evaluations, and for varying numbers of departments and respondents per department for hospital-level evaluations.

|  | *Department evaluations* | | | *Hospital evaluations* | | | |
| --- | --- | --- | --- | --- | --- | --- | --- |
| *Subscale* | *N respondents* | *G coeff* | *SEM* | *N departments* | *N respondents /department* | *G coeff* | *SEM* |
| *1. Admission* | 10 | 0.37 | 0.077 | 2 | 20 | 0.08 | 0.056 |
|  | 20 | 0.54 | 0.054 | 4 | 20 | 0.15 | 0.040 |
|  | 30 | 0.64 | 0.044 | 6 | 20 | 0.20 | 0.032 |
|  | 40 | 0.70 | 0.038 | 8 | 20 | 0.26 | 0.028 |
|  | 50 | 0.75 | 0.034 | 10 | 20 | 0.30 | 0.025 |
|  | 60 | 0.78 | 0.031 | 2 | 50 | 0.11 | 0.048 |
|  | 70 | 0.81 | 0.029 | 4 | 50 | 0.19 | 0.034 |
|  | 80 | 0.83 | 0.027 | 6 | 50 | 0.27 | 0.027 |
|  | 90 | 0.84 | 0.026 | 8 | 50 | 0.32 | 0.024 |
|  | 100 | 0.86 | 0.024 | 10 | 50 | 0.38 | 0.021 |
|  | 150 | 0.90 | 0.020 | 10 | 100 | 0.41 | 0.020 |
| *2. Communication with nurses* | 10 | 0.19 | 0.190 | 2 | 20 | 0.27 | 0.106 |
|  | 20 | 0.32 | 0.134 | 4 | 20 | 0.43 | 0.075 |
|  | 30 | 0.41 | 0.110 | 6 | 20 | 0.53 | 0.061 |
|  | 40 | 0.49 | 0.095 | 8 | 20 | 0.60 | 0.053 |
|  | 50 | 0.54 | 0.085 | 10 | 20 | 0.65 | 0.047 |
|  | 60 | 0.59 | 0.078 | 2 | 50 | 0.42 | 0.077 |
|  | 70 | 0.62 | 0.072 | 4 | 50 | 0.59 | 0.054 |
|  | 80 | 0.65 | 0.067 | 6 | 50 | 0.68 | 0.044 |
|  | 90 | 0.68 | 0.063 | 8 | 50 | 0.74 | 0.038 |
|  | 100 | 0.70 | 0.060 | 10 | 50 | 0.78 | 0.034 |
| *3. Communication with doctors* | 10 | 0.17 | 0.221 | 2 | 20 | 0.19 | 0.125 |
|  | 20 | 0.29 | 0.157 | 4 | 20 | 0.32 | 0.088 |
|  | 30 | 0.38 | 0.128 | 6 | 20 | 0.41 | 0.072 |
|  | 40 | 0.45 | 0.111 | 8 | 20 | 0.49 | 0.062 |
|  | 50 | 0.50 | 0.099 | 10 | 20 | 0.54 | 0.056 |
|  | 60 | 0.55 | 0.090 | 2 | 50 | 0.31 | 0.090 |
|  | 70 | 0.59 | 0.084 | 4 | 50 | 0.47 | 0.064 |
|  | 80 | 0.62 | 0.078 | 6 | 50 | 0.57 | 0.052 |
|  | 90 | 0.65 | 0.073 | 8 | 50 | 0.64 | 0.045 |
|  | 100 | 0.67 | 0.070 | 10 | 50 | 0.69 | 0.040 |
| *4. Own contribution* | 10 | 0.45 | 0.201 | 2 | 20 | 0.54 | 0.131 |
|  | 20 | 0.62 | 0.142 | 4 | 20 | 0.70 | 0.092 |
|  | 30 | 0.71 | 0.116 | 6 | 20 | 0.78 | 0.075 |
|  | 40 | 0.77 | 0.101 | 8 | 20 | 0.83 | 0.065 |
|  | 50 | 0.80 | 0.090 | 10 | 20 | 0.86 | 0.058 |
|  | 60 | 0.83 | 0.082 | 2 | 50 | 0.65 | 0.105 |
|  | 70 | 0.85 | 0.076 | 4 | 50 | 0.79 | 0.074 |
|  | 80 | 0.87 | 0.071 | 6 | 50 | 0.85 | 0.061 |
|  | 90 | 0.88 | 0.067 | 8 | 50 | 0.88 | 0.052 |
|  | 100 | 0.89 | 0.064 | 10 | 50 | 0.90 | 0.047 |
| *5. Explanation of treatment* | 10 | 0.26 | 0.21 | 2 | 20 | 0.17 | 0.130 |
|  | 20 | 0.41 | 0.15 | 4 | 20 | 0.29 | 0.092 |
|  | 30 | 0.51 | 0.12 | 6 | 20 | 0.38 | 0.075 |
|  | 40 | 0.58 | 0.1 | 8 | 20 | 0.45 | 0.065 |
|  | 50 | 0.63 | 0.09 | 10 | 20 | 0.50 | 0.058 |
|  | 60 | 0.68 | 0.09 | 2 | 50 | 0.25 | 0.102 |
|  | 70 | 0.71 | 0.08 | 4 | 50 | 0.40 | 0.072 |
|  | 80 | 0.74 | 0.07 | 6 | 50 | 0.50 | 0.059 |
|  | 90 | 0.76 | 0.07 | 8 | 50 | 0.57 | 0.051 |
|  | 100 | 0.78 | 0.07 | 10 | 50 | 0.62 | 0.045 |
| *6. Pain management* | 10 | 0.21 | 0.194 | 2 | 20 | 0.14 | 0.115 |
|  | 20 | 0.34 | 0.137 | 4 | 20 | 0.25 | 0.081 |
|  | 30 | 0.44 | 0.112 | 6 | 20 | 0.33 | 0.067 |
|  | 40 | 0.51 | 0.097 | 8 | 20 | 0.39 | 0.058 |
|  | 50 | 0.57 | 0.087 | 10 | 20 | 0.45 | 0.052 |
|  | 60 | 0.61 | 0.079 | 2 | 50 | 0.22 | 0.087 |
|  | 70 | 0.65 | 0.073 | 4 | 50 | 0.36 | 0.062 |
|  | 80 | 0.68 | 0.069 | 6 | 50 | 0.46 | 0.050 |
|  | 90 | 0.70 | 0.065 | 8 | 50 | 0.53 | 0.044 |
|  | 100 | 0.72 | 0.061 | 10 | 50 | 0.59 | 0.039 |
| *7. Communication about medication* | 10 | 0.19 | 0.284 | 2 | 20 | 0.22 | 0.162 |
|  | 20 | 0.32 | 0.201 | 4 | 20 | 0.37 | 0.114 |
|  | 30 | 0.42 | 0.164 | 6 | 20 | 0.47 | 0.093 |
|  | 40 | 0.49 | 0.142 | 8 | 20 | 0.54 | 0.081 |
|  | 50 | 0.54 | 0.127 | 10 | 20 | 0.59 | 0.072 |
|  | 60 | 0.59 | 0.116 | 2 | 50 | 0.35 | 0.119 |
|  | 70 | 0.63 | 0.107 | 4 | 50 | 0.52 | 0.084 |
|  | 80 | 0.66 | 0.100 | 6 | 50 | 0.62 | 0.068 |
|  | 90 | 0.68 | 0.095 | 8 | 50 | 0.68 | 0.059 |
|  | 100 | 0.71 | 0.090 | 10 | 50 | 0.73 | 0.053 |
| *8. Feeling of safety* | 10 | 0.22 | 0.211 | 2 | 20 | 0.12 | 0.127 |
|  | 20 | 0.36 | 0.149 | 4 | 20 | 0.21 | 0.090 |
|  | 30 | 0.45 | 0.122 | 6 | 20 | 0.29 | 0.074 |
|  | 40 | 0.52 | 0.106 | 8 | 20 | 0.35 | 0.064 |
|  | 50 | 0.58 | 0.094 | 10 | 20 | 0.40 | 0.057 |
|  | 60 | 0.62 | 0.086 | 2 | 50 | 0.19 | 0.098 |
|  | 70 | 0.66 | 0.080 | 4 | 50 | 0.32 | 0.069 |
|  | 80 | 0.69 | 0.075 | 6 | 50 | 0.41 | 0.056 |
|  | 90 | 0.71 | 0.070 | 8 | 50 | 0.48 | 0.049 |
|  | 100 | 0.73 | 0.067 | 10 | 50 | 0.54 | 0.044 |
| *9. Information at discharge* | 10 | 0.37 | 0.094 | 2 | 20 | 0.09 | 0.068 |
|  | 20 | 0.54 | 0.067 | 4 | 20 | 0.17 | 0.048 |
|  | 30 | 0.64 | 0.054 | 6 | 20 | 0.23 | 0.039 |
|  | 40 | 0.70 | 0.047 | 8 | 20 | 0.29 | 0.034 |
|  | 50 | 0.75 | 0.042 | 10 | 20 | 0.33 | 0.030 |
|  | 60 | 0.78 | 0.038 | 2 | 50 | 0.12 | 0.057 |
|  | 70 | 0.81 | 0.036 | 4 | 50 | 0.22 | 0.041 |
|  | 80 | 0.83 | 0.033 | 6 | 50 | 0.30 | 0.033 |
|  | 90 | 0.84 | 0.031 | 8 | 50 | 0.36 | 0.029 |
|  | 100 | 0.86 | 0.030 | 10 | 50 | 0.41 | 0.026 |
|  | 150 | 0.90 | 0.024 | 10 | 100 | 0.45 | 0.024 |
